# Adjuvant Hepatic Arterial Infusion Pump Chemotherapy After Resection of Colorectal Liver Metastases: Results of a Safety and Feasibility Study in The Netherlands

**DOI:** 10.1245/s10434-019-07973-w

**Published:** 2019-10-22

**Authors:** Florian E. Buisman, Dirk J. Grünhagen, Marjolein Y. V. Homs, Cecile Grootscholten, Wills F. Filipe, Nancy E. Kemeny, Andrea Cercek, Micheal I. D’Angelica, Maarten L. Donswijk, Leni van Doorn, Jasper Emmering, William R. Jarnagin, T. Peter Kingham, Elisabeth G. Klompenhouwer, Niels F. M. Kok, Maria C. Kuiper, Adriaan Moelker, Warner Prevoo, Michelle W. J. Versleijen, Cornelis Verhoef, Koert F. D. Kuhlmann, Bas Groot Koerkamp

**Affiliations:** 1 Department of Surgery, Erasmus MC Cancer Institute, Erasmus University, Rotterdam, The Netherlands; 2 Department of Medical Oncology, Erasmus MC Cancer Institute, Erasmus University, Rotterdam, The Netherlands; 3 Department of Medical Oncology, The Netherlands Cancer Institute, Amsterdam, The Netherlands; 4 Department of Medical Oncology, Memorial Sloan Kettering Cancer Center, New York, NY USA; 5 Department of Surgery, Memorial Sloan Kettering Cancer Center, New York, NY USA; 6 Department of Nuclear Medicine, The Netherlands Cancer Institute, Amsterdam, The Netherlands; 7 Department of Radiology and Nuclear Medicine, Erasmus MC, Erasmus University, Rotterdam, The Netherlands; 8 Department of Radiology, The Netherlands Cancer Institute, Amsterdam, The Netherlands; 9 Department of Surgery, The Netherlands Cancer Institute, Amsterdam, The Netherlands

## Abstract

**Background:**

The 10-year overall survival with adjuvant hepatic arterial infusion pump (HAIP) chemotherapy after resection of colorectal liver metastases (CRLMs) was 61% in clinical trials from Memorial Sloan Kettering Cancer Center. A pilot study was performed to evaluate the safety and feasibility of adjuvant HAIP chemotherapy in patients with resectable CRLMs.

**Study Design:**

A phase II study was performed in two centers in The Netherlands. Patients with resectable CRLM without extrahepatic disease were eligible. All patients underwent complete resection and/or ablation of CRLMs and pump implantation. Safety was determined by the 90-day HAIP-related postoperative complications from the day of pump placement (Clavien–Dindo classification, grade III or higher) and feasibility by the successful administration of the first cycle of HAIP chemotherapy.

**Results:**

A total of 20 patients, with a median age of 57 years (interquartile range [IQR] 51–64) were included. Grade III or higher HAIP-related postoperative complications were found in two patients (10%), both of whom had a reoperation (without laparotomy) to replace a pump with a slow flow rate or to reposition a flipped pump. No arterial bleeding, arterial dissection, arterial thrombosis, extrahepatic perfusion, pump pocket hematoma, or pump pocket infections were found within 90 days after surgery. After a median of 43 days (IQR 29–52) following surgery, all patients received the first dose of HAIP chemotherapy, which was completed uneventfully in all patients.

**Conclusion:**

Pump implantation is safe, and administration of HAIP chemotherapy is feasible, in patients with resectable CRLMs, after training of a dedicated multidisciplinary team.

Recurrent disease is reported in up to 70% of patients after resection of colorectal liver metastases (CRLMs).^1^ Reported 5- and 10-year overall survival (OS) of CRLM patients treated with resection and systemic chemotherapy were 40% and 25%, respectively.^1^

The rationale of adjuvant hepatic arterial infusion pump (HAIP) chemotherapy after resection of CRLM is that initial recurrences involve the liver in half of the patients. HAIP chemotherapy involves a subcutaneous surgically implanted pump that delivers chemotherapy through a catheter directly into the hepatic artery via the gastroduodenal artery (GDA). Arterial administration is preferred because liver tumors mainly depend on arterial rather than portal venous blood supply.^2^^,^^3^ Floxuridine (or FUDR) is the preferred drug for HAIP chemotherapy. Due to its high hepatic extraction rate, intratumoral exposure is up to 400-fold higher compared with systemic administration, with little or no systemic toxicity.^4^ Two randomized controlled trials (RCTs) performed in the 1990s demonstrated superior OS of HAIP chemotherapy. Moreover, the 10-year OS of patients who received adjuvant HAIP chemotherapy in several phase II trials after 2003 was 61%.^5^^,^^6^

Regardless of these impressive results, HAIP chemotherapy is not commonly used outside of Memorial Sloan Kettering Cancer Center (MSKCC). One of the barriers is that floxuridine is not registered in the European Union (EU). Moreover, HAIP chemotherapy requires comprehensive training of and commitment from a multidisciplinary team.

Previous studies demonstrated both safety and feasibility concerns due to its complexity, requiring both technical knowledge and practical skills.^7^^–^9 However, a previous study of 544 patients demonstrated that an experienced team was associated with less pump-related complications. The pump failure rate was only 5% in the first 6 months after implantation.^2^ HAIP-related postoperative complications include pump flow-rate abnormalities, pump dislocation, arterial bleeding, arterial dissection, arterial thrombosis, extrahepatic perfusion, pump pocket hematoma, and pump pocket infections.^8^

The aim of this study was to determine the safety and feasibility of adjuvant HAIP chemotherapy after resection of CRLMs in two centers in The Netherlands.

## Methods

### Study Design

A phase II, multicenter, single-arm, safety and feasibility study was conducted from February 2018 to February 2019 at the Erasmus MC Cancer Institute (Rotterdam) and The Netherlands Cancer Institute (Amsterdam) in The Netherlands. The Institutional Review Board approved the study protocol (MEC-2017-282). The study was registered in the Netherlands Trial Register (number 6917).

### Patients

All patients with histologically confirmed colorectal cancer and resectable CRLM without extrahepatic disease (EHD) were evaluated for inclusion. EHD was defined as any disease outside the liver prior to or at time of diagnosis of CRLM. Patients with EHD found at surgery were excluded. Patients were also excluded if positioning of a catheter for HAIP chemotherapy was not feasible based on a preoperative arterial computed tomography (CT) scan, prior hepatic radiation or resection, CRLM requiring two-staged resection, liver-first approach, and diagnosis of another malignancy. Positioning of a catheter was not considered feasible if the GDA had no connecting branch to the left or right liver (e.g. in a patient with a completely replaced right and left hepatic artery).

### Training

The initial eight implantations (four in each center) were performed under the supervision of surgeons from MSKCC (MD and TK), who both have over 10-years of experience in pump implantations (i.e. a total of more than 200 implantations each). The multidisciplinary teams of both participating centers visited MSKCC for a 2-day workshop. Additional training involved detailed protocols and video material of the surgical pump implantation. A PhD student attended all implantations, supervised pump refills, and provided hands-on workshops for nurses and staff members.

### Surgical Procedure

A dedicated team of two surgeons in each center performed all implantations. Surgical resection of CRLMs by laparotomy, with or without resection of the primary tumor, was combined with implantation of the HAI pump with a constant non-programmable flow rate (Tricumed IP2000 V). This pump has similar specifications as the pump used in MSKCC (Codman 3000), with the main difference being that the reservoir of the Tricumed pump is pressurized by butane rather than Freon, which is not allowed in the EU due to environmental laws. Treatment of the CRLMs involved complete resection and/or open ablation. In case of a simultaneous resection of the primary tumor, liver resection with pump implantation was performed first, followed by resection of the primary tumor to prevent contamination of the pump. Prior to pump implantation, a function test was performed to check the adequate operation of the pump. A cholecystectomy was performed to avoid cholecystitis as a result of intra-arterial chemotherapy through the cystic artery.^10^ The pump pocket was created at the left-lower quadrant of the abdominal wall, or in the right-lower quadrant in patients with a colostomy. The pocket cavity was created three-quarters caudal to the incision to ensure easy access of the pump septum for percutaneous refills.

The entire GDA, as well as the proximal proper hepatic artery, were mobilized and dissected circumferentially from their attachments to facilitate insertion of the catheter and to avoid inadvertent perfusion of the pancreas, stomach, or duodenum. The distal GDA was ligated with a non-absorbable tie and a transversal arteriotomy was performed followed by insertion of the catheter. The catheter was positioned just at the origin of the GDA. Positioning of the catheter with the tip in the hepatic artery may cause turbulence and the risk of thrombosis, while positioning the catheter too far from the hepatic artery may cause pooling of floxuridine in the GDA, with a risk of erosion, a pseudoaneurysm, and hemorrhage. A metal connector was used to connect a commercially available (B. Braun Celsite^®^) intra-arterial catheter (distal catheter) with the Tricumed catheter that comes with the pump (Tricumed Catheter 1000^®^). This connection was secured with two non-absorbable ties. The distal catheter has several beads (i.e. local thickening of the catheter wall), which were used to secure the catheter with non-absorbable ties in the GDA. Perfusion of both lobes of the liver and lack of extrahepatic perfusion was confirmed by an intraoperative bolus injection of methylene blue. After the perfusion test, the catheter was flushed with heparinized saline, and the wounds were closed. Any replaced and accessory hepatic arteries were ligated, provided that a patent GDA connected with at least one hepatic artery was present. Intrahepatic shunts will typically reassure that the catheter perfuses all liver segments, which was confirmed intraoperatively, and during follow-up with postoperative scintigraphy.

### Postoperative Procedures

Prior to the start of HAIP chemotherapy, a postoperative technetium-99-labeled macroaggregated albumin (Tc-99m MAA) scintigraphy was performed to again confirm the absence of extrahepatic perfusion. In case of extrahepatic perfusion, patients were evaluated angiographically and branches were embolized with re-testing prior to the start of treatment.

### Chemotherapeutic Regimen

HAIP chemotherapy was initiated 4–12 weeks after surgery depending on the patients’ condition and liver function. The pump was refilled with a heparinized saline solution (35,000 IE in 35 mL NaCl 0.9%) every 2 weeks until the start of HAIP chemotherapy to prevent thrombosis of the catheter. All patients were scheduled for six cycles of 4 weeks of HAIP chemotherapy with floxuridine. Each cycle comprised 2 weeks of HAIP chemotherapy followed by a 2-week rest period during which the pump was filled with the heparinized saline solution. Floxuridine was administered based on the MSKCC regimen (0.12 mg/kg/day).^11^^,^^12^ If the actual weight was more than 25% above the ideal weight, the dose of floxuridine was calculated using the average of the actual and ideal weight. Floxuridine was administered in a solution of 35,000 IE heparin and 25 mg dexamethasone in NaCl 0.9% with a total volume of 35 ml. A prophylactic dose of 20 mg of proton pump inhibitors was administered daily during HAIP chemotherapy. No adjuvant systemic chemotherapy was administered since this is not the standard of care in The Netherlands.

### Outcomes

Safety was determined by the percentage of postoperative complications (Clavien–Dindo classification grade III or higher) within 90 days after surgery related to HAI pump placement. The feasibility was defined as the percentage of patients receiving at least one cycle of adjuvant HAIP chemotherapy after resection of CRLMs.

### Definitions and Statistical Analysis

Demographic and clinicopathological characteristics were presented as medians with interquartile ranges (IQRs), and as means with ranges for continuous variables and proportions for categorical variables. CRLMs that were detected within 3 months of resection of the primary tumor were considered synchronous. Any chemotherapy administered within 3 months prior to resection was considered as preoperative chemotherapy. A positive resection margin (R1) was defined as tumor cells present at the resection margin, and major liver resection was defined as complete resection of three or more segments. All analyses were performed using SPSS version 24 (IBM Corporation, Armonk, NY, USA).

## Results

A total of 22 patients were included in two centers (Erasmus MC Cancer Institute and The Netherlands Cancer Institute, The Netherlands) from February 2018 until February 2019. Two patients were excluded during surgery; one patient had unresectable CRLMs found during intraoperative ultrasonography, and one patient was excluded due to an occult peritoneal lesion that was found during surgery and confirmed by frozen section biopsy. No patients were excluded due to unexpected abnormal hepatic artery anatomy. A total of 20 patients were eligible.

### Baseline Characteristics

Baseline characteristics of 20 patients who were eligible for surgical treatment and pump implantation are shown in Table 1. Median age was 57 years (IQR 51–64), and the majority of patients were male (*n* = 12, 60%). Most patients had left-sided colorectal cancer (*n* = 11, 55%), followed by rectal (*n* = 6, 30%) and right-sided colorectal cancer (*n* = 3, 15%). About half of the patients had synchronous CRLMs (*n* = 11, 55%).

**Table 1:** Baseline characteristics

	Age (years)	Sex	ASA score	BMI (kg/m^2^)	Location CRC	T-stage CRC	Nodal status	Synchronous/metachronous	DFI (months)	No. of CRLMs	Size of the largest CRLM (cm)	CEA (µg/L)
Median (IQR) or *n* (%)	57 (51–64)	Male: 12 (60)	2 (1–2)	27 (24–27)	Right: 3 (15)	3 (3–4)	N0: 6 (30)	Syn: 11 (55)	1 (0–13)	2 (1–5)	2.3 (0.8–7.1)	6 (3–26)
Case 1	58	Female	3	27	Rectum	3	0	Metachronous	6	1	6.7	23
Case 2	64	Male	2	34	Left	3	2	Metachronous	41	3	2.4	6
Case 3	52	Female	2	29	Left	3	1	Metachronous	13	1	2.8	5
Case 4	64	Male	3	24	Left	3	1	Metachronous	13	1	1.8	5
Case 5	75	Female	2	26	Left	3	0	Synchronous	0	1	2.2	8
Case 6	67	Male	1	24	Left	4	0	Synchronous	0	2	4.8	34
Case 7	50	Female	1	24	Left	3	0	Synchronous	2	2	2.0	63
Case 8	54	Male	3	24	Right	4	2	Metachronous	28	2	2.3	27
Case 9	68	Male	2	24	Right	4	2	Synchronous	0	5	1.2	40
Case 10	58	Male	2	25	Left	3	1	Synchronous	0	5	7.1	2
Case 11	57	Female	2	25	Left	4	2	Metachronous	15	2	4.8	1
Case 12	66	Male	3	23	Left	4	1	Synchronous	0	4	4.2	24
Case 13	51	Female	1	27	Rectum	3	1	Metachronous	2	5	1.3	3.
Case 14	42	Female	1	23	Left	3	0	Metachronous	4	13	1.5	3
Case 15	54	Female	1	25	Right	3	1	Synchronous	0	3	2.5	19
Case 16	61	Male	2	25	Left	3	1	Synchronous	0	2	1.6	3
Case 17	54	Male	2	28	Rectum	3	1	Synchronous	0	7	52	880
Case 18	43	Male	1	25	Rectum	2	1	Synchronous	0	12	0.8	4
Case 19	57	Male	1	24	Rectum	3	0	Metachronous	12	2	2.2	6
Case 20	46	Male	2	24	Rectum	2	1	Synchronous	0	1	1.0	3

*ASA* American Society of Anesthesiologists, *BMI* body mass index, *CEA* carcinoembryonic antigen, *CRC* colorectal cancer, *CRLM* colorectal liver metastasis, *DFI* disease-free interval, *IQR* interquartile range, *N0* node-negative, *Syn* synchronous, *T*-*stage CRC* tumor-stage colorectal cancer

### Surgical Aspects

Surgical aspects are summarized in Table 2. Preoperative chemotherapy was administered in seven patients (35%). In four patients (20%), the procedure was combined with simultaneous resection of the primary tumor; in seven patients (35%), ablation was combined with resection; and in two patients (10%), only open ablation and pump implantation were performed. A major liver resection was performed in four patients (20%). The hepatic arterial anatomy was abnormal in 10 patients (50%), requiring ligation of accessory or replaced left and/or right hepatic arteries. The pump was positioned in the left-lower quadrant of the abdomen in 18 patients (90%). The right-lower abdomen was the preferred site in two patients (10%) due to a prior colostomy in the left-lower quadrant. The median hospital stay was 8 days (IQR 6–9). Postoperative Tch-99m MAA scintigraphy showed no signs of extrahepatic perfusion in all patients.

**Table 2:** Surgical outcomes

	Procedure	Preoperative CTx	Resection/ablation	Major resection	Hepatic arterial anatomy	Resection margin	Operative time (min)	Blood loss (mL)	Hospital stay (days)	Days to start HAIP
Median (IQR) or *n*(%)	Primary first: 4 (20)	Yes: 7 (35)	Resection only: 11 (55)	Yes: 4 (20)	Abnormal: 10 (50)	R1: 4 (20)	226 (187–290)	610 (200–838)	8 (6–9)	43 (29–52)
Case 1	Primary first	No	Resection	No	Normal	R0	169	0	7	41
Case 2	Primary first	No	Resection	No	Abnormal	R1	204	1360	6	55
Case 3	Primary first	No	Ablation only	No	Normal	Ablation	161	0	6	54
Case 4	Primary first	Yes	Resection	No	Abnormal	R1	176	280	7	47
Case 5	Simultaneous^a^	No	Resection	No	Normal	R0	204	560	6	28
Case 6	Simultaneous^a^	Yes	Resection	No	Abnormal	R0	351	2850	31	56
Case 7	Primary first	No	Resection + ablation	Yes	Abnormal	R0	246	800	9	43
Case 8	Primary first	No	Resection	No	Normal	R0	180	710	10	43
Case 9	Primary first	Yes	Resection	No	Abnormal	R1	283	2000	7	41
Case 10	Primary first	Yes	Resection	Yes	Normal	R0	331	1700	8	57
Case 11	Primary first	Yes	Resection	Yes	Normal	R0	241	750	9	55
Case 12	Simultaneous^a^	No	Resection + ablation	No	Normal	R0	316	0	9	42
Case 13	Primary first	No	Resection + ablation	No	Normal	R0	315	620	6	26
Case 14	Primary first	Yes	Resection + ablation	Yes	Abnormal	R0	267	600	9	29
Case 15	Primary first	Yes	Resection	No	Normal	R1	188	650	5	29
Case 16	Primary first	No	RFA only	No	Abnormal	Ablation	206	200	4	43
Case 17	Simultaneous^b^	No	Resection + ablation	No	Abnormal	R0	287	850	9	43
Case 18	Primary first	No	Resection + ablation	No	Abnormal	R0	291	150	7	29
Case 19	Primary first	No	Resection + ablation	No	Normal	R0	186	200	9	27
Case 20	Primary first	No	Resection	No	Normal	R0	210	200	8	27

*CTx* chemotherapy, *HAIP* hepatic arterial infusion pump, *IQR* interquartile range, *R1* irradical resection margin, *RFA* radiofrequency ablation

^a^Sigmoid resection

^b^Low anterior resection

### Postoperative Complications

Postoperative complications are summarized in Table 3. No postoperative 90-day mortality was found. No arterial bleeding, arterial dissection, arterial thrombosis, pump pocket hematoma, or pump pocket infections were found within the first 90 days after surgery. Five patients (25%) had postoperative complications of grade III or higher. Two patients (10%) had complications related to HAI pump placement; the first patient required pump replacement due to a decreased flow rate of the pump, and the second patient had a flipped pump (upside down) requiring reoperation. In both patients, reoperation involved a local exploration of the pump pocket without a laparotomy, with same-day discharge. Both patients recovered uneventful and continued HAIP chemotherapy within 2 weeks.

**Table 3:** Postoperative complications within 90 days of surgery

Number	Grade III or higher (Clavien–Dindo)	HAI pump-related	Time to event (days)	Requiring readmission	Requiring surgery	Specified
Total (%)	5 (25)	2 (10)		4 (20)	3 (15)	
Case 1	–					
Case 2	IIIb	Yes	42	Yes	Yes	Pump replacement due to slow flow rate
Case 3	IIIb	Yes	41	Yes	Yes	Flipped pump
Case 4	–					
Case 5	–					
Case 6	IVa	No	4	No	Yes (2×)	1. Biliary peritonitis due to biliary leakage at the liver resection margin 2. Threatening abdominal fascial dehiscence
Case 7	–					
Case 8	–					
Case 9	–					
Case 10	IIIa	No	13	Yes	No	Percutaneous drainage of sterile abdominal fluid collection
Case 11	IIIa	No	9	Yes	No	Spontaneous bacterial peritonitis requiring antibiotics and percutaneous drainage
Case 12	–					
Case 13	–					
Case 14	–					
Case 15	–					
Case 16	–					
Case 17	–					
Case 18	–					
Case 19	–					
Case 20	–					

*HAI* hepatic arterial infusion

Another three patients (15%) required re-interventions due to complications unrelated to HAI pump implantation. One patient required a re-laparotomy for biliary peritonitis as a result of biliary leakage at the liver resection margin, as well as a second re-laparotomy due to fascial dehiscence. A second patient was readmitted with an intra-abdominal fluid collection that was treated with both percutaneous drainage and intravenous antibiotics. The third patient was readmitted for percutaneous drainage of an intra-abdominal fluid collection with negative culture. All three patients recovered uneventful.

### Initiation of Hepatic Arterial Infusion Pump (HAIP) Chemotherapy

The median period to administration of the first cycle of HAIP chemotherapy was 43 days (IQR 29–52). Percutaneous access of the pump for the first cycle of HAIP chemotherapy was performed without adverse events in all patients. All patients uneventfully completed the first cycle of HAIP chemotherapy, which was the primary endpoint for feasibility.

## Discussion

This study demonstrated that HAI pump implantation and administration of adjuvant HAIP chemotherapy in patients with resectable CRLMs is safe and feasible in The Netherlands. Safety was demonstrated as two patients (10%) developed HAIP-related postoperative complications that were resolved with a reoperation to replace or reposition the subcutaneous pump. Feasibility was demonstrated because all patients started HAIP chemotherapy within 6 weeks after surgery.

As a result of the pump with a decreased flow rate, the pre-implantation pump performance test procedure was adapted. The pump flow rate is temperature-dependent, reaching optimal flow rates at body temperature. The new pump performance test included continuous heating of the pump to 37 °C within an ex vivo heater, allowing precise observation of pump flow rate mimicking in vivo conditions. In order to minimize the risk of pump dislocation (flipping within the pump pocket), all pockets were created with minimal residual space in order to achieve a tight fit with minimal risk of dislocation of the infusion pump in the pocket. In obese patients (i.e. BMI > 30) we prefer to position the pump on the chest wall. The observed complication rate seems acceptable compared with a large retrospective study in which 544 patients who underwent pump implantation for CRLMs were evaluated.^8^ Pump-related complications were reported in 120 patients (22%) and were classified as related to the hepatic arterial system (*n* = 62, 51%), the catheter (*n* = 33, 26%), the pump-pocket (*n* = 19, 16%), or the pump (*n* = 6, 5%). Technical complications could be salvaged in 54 patients (45%). A higher rate of complications was found with surgeons who performed less than 25 implantations (31% vs. 19%; *p* < 0.001). Other perioperative factors were comparable with our study: mean operative time (260 min vs. 241 min in our study), mean blood loss (490 mL vs. 724 mL in our study), and length of hospital stay (8 days vs. 9 days in our study). However, long-term follow-up is needed for complete comparison of our results with this study.

Our multidisciplinary approach with extensive training and proctoring by MSKCC was essential for the safety and feasibility of setting up an HAIP chemotherapy program. In a previous RCT on hepatic arterial infusion (HAI) chemotherapy, lack of training and experience appeared to be the major factor for the failure of safety and feasibility.^7^ Lorenz et al. compared resection of CRLMs combined with adjuvant HAI of 5-fluorouracil, with resection of CRLM alone. The trial was prematurely terminated after interim analysis for futility. At the time of interim analysis, a total of 113 patients were randomized into each group. Eight patients (7%) within the HAI group died within 30 days after surgery; four deaths were related to HAI chemotherapy toxicity, three were related to catheter-related bleeding, and one was related to to angiography-induced shock. In the control group, three patients (3%) died within 30 days. High rates of dropouts, i.e. patients who did not receive the assigned treatment, were reported in both groups, with various reasons: 24 patients (21%) assigned to HAI + resection (no catheter implanted [*n* = 7], CRLM not resected [*n* = 6], malperfusion [*n* = 5], refusal of patient [*n* = 2], port complications [*n* = 2], liver cirrhosis [*n *= 1], and postoperative ileus [*n* = 1]), and 13 patients (12%) assigned to resection alone (CRLM not resected [*n* = 10] and residual disease after resection [*n* = 3]). Explanations that could have accounted for the failure of this trial included participation of 26 centers, with each center performing only approximately one intra-arterial catheter placement per year; the use of a port with a catheter in the flow of the hepatic artery, resulting in a high rate of technical failures (e.g. hepatic arterial thrombosis); and the use of intra-arterial 5-fluorouracil, which is not only less effective (lower dose due to a smaller first-pass effect) but also has a much higher systemic exposure compared with floxuridine.^4^

Several studies, including an RCT, demonstrated superior survival of HAIP chemotherapy compared with systemic chemotherapy alone in patients with resectable CRLM.^5^^,^^6^^,^^13^^,^^14^ A phase III RCT for adjuvant HAIP chemotherapy found an improvement in 2-year survival (86% vs. 72%; *p* = 0.03).^13^ Long-term follow-up of 287 patients receiving adjuvant HAIP chemotherapy in four prospective trials at MSKCC demonstrated a 10-year OS of 61%.^5^ A recent propensity scored analysis demonstrated an OS benefit of 23 months of adjuvant HAIP chemotherapy compared with systemic chemotherapy alone (67 months vs. 44 months; *p* < 0.001).^14^ The OS without HAIP was similar with other large cohorts outside MSKCC.^15^ The difference remained at propensity score analysis, with an adjusted hazard ratio of 0.67 (95% confidence interval 0.59–0.76; *p* < 0.001).

Implementation of HAIP chemotherapy beyond MSKCC is currently limited to a few centers around the world (e.g. University Hospital Zurich, Zurich, Switzerland; University of Pittsburgh Medical Center, Pittsburgh, PA, USA; and Washington University School of Medicine, St. Louis, MO, USA). Several explanations have been suggested that could account for this. The historical perspective may be partly responsible. The first trials on HAIP chemotherapy date from the 1990s, a decade in which new promising agents for systemic chemotherapy such as irinotecan and oxaliplatin were introduced. Administration of intravenous drugs was simple compared with the implementation of HAIP chemotherapy, which required new skills and close collaboration within multidisciplinary teams. Modern systemic chemotherapy results in superior survival in selected patients with stage IV CRC.^16^^,^^17^ In the subgroup of patients with resectable CRLM, no OS benefit was found (*p* = 0.30) in a phase III RCT, although progression-free survival was superior in the per protocol analysis (*p* = 0.035).^18^ Despite these results, the recurrence rate was still about 70%. This disappointing high percentage of recurrent disease after curative resection of CRLMs and perioperative systemic chemotherapy has renewed interest in adjuvant HAIP chemotherapy.

Second, regulatory factors have also opposed implementation of HAIP chemotherapy outside the US. Floxuridine was first registered by the US FDA in 1971; however, in the EU, floxuridine cannot be used outside clinical trials since it is not registered. Others have resorted to 5-fluorouracil or oxaliplatin instead of floxuridine. A previous trial on intermittent infusion of 5-fluorouracil through a mediport in the hepatic artery was terminated prematurely, mainly due to a high rate of 5-fluorouracil-related complications.^7^ However, the hallmark of HAIP chemotherapy is the 95% first-pass effect of floxuridine in the liver that allows for a very high dosage with continuous infusion without systemic toxicity.

No infusion pump with the intended use of intra-arterial chemotherapy is currently registered in the EU. The Tricumed IP2000 V infusion pump is CE marked and has been used for many years in patients with spasticity and chronic pain. Both registration of floxuridine, and an infusion pump for HAIP chemotherapy, are essential steps for implementation of this treatment in the EU. The subcutaneous pump is a key component of the intra-arterial chemotherapy because floxuridine has a half-life of only 10 min.^4^ A percutaneous approach for the delivery of intra-arterial chemotherapy has been investigated by the Gustave Roussy Hospital in Paris (France).^19^ Goéré et al. administered intra-arterial oxaliplatin using a percutaneous catheter in the hepatic artery. With the percutaneous approach, a catheter remains positioned in the flow of the hepatic artery, with a higher risk of hepatic artery thrombosis. Therefore, the percutaneous approach is not suitable for prolonged administration. The pump has an intra-arterial catheter in the GDA, outside the hepatic arterial flow and therefore less likely to cause thrombosis. The pump can stay in for many years for the treatment of disease recurrence in the liver. Furthermore, the surgical approach allows complete circumferential dissection of the artery, which is important to avoid complications of extrahepatic perfusion of floxuridine. The potential effects of extrahepatic perfusion of floxuridine are more severe than the effects of extrahepatic perfusion of oxaliplatin due to the high dose of floxuridine administered compared with oxaliplatin, provided by its high first-pass effect.

## Conclusions

This is the first study prospectively reporting early safety and feasibility results on adjuvant HAIP chemotherapy in patients with resectable CRLM. Some fundamental elements have been considered in the design of our program, including thorough training on all safety and technical aspects, selection of appropriate materials, and careful selection of patients and participating centers. The number of participating centers for the safety and feasibility study was only two, to guarantee adequate training and experience. All future implantations will be performed by a team of two experienced surgeons, and new surgeons will only be allowed to perform implantations after thorough training to sustain knowledge and skills.

After confirming safety and feasibility, we proceeded with a multicenter, phase III RCT (the PUMP trial) to study the effectiveness of adjuvant HAIP chemotherapy in patients with resectable CRLMs (www.trialregister.nl: NTR7493).^20^

This study showed that starting an HAIP chemotherapy program can be safe and feasible after adequate training and proctoring of a multidisciplinary team.
